# Comprehensive Facial Reconstruction With Anterolateral Thigh Free Flap After Maxillary Sinus Squamous Cell Carcinoma Resection

**DOI:** 10.7759/cureus.96508

**Published:** 2025-11-10

**Authors:** Bhathiya Nakandala, Pragaash Shanmuganathan, Dasith K Jayawickrama

**Affiliations:** 1 Surgery, National Hospital of Sri Lanka, Colombo, LKA; 2 Plastic and Reconstructive Surgery, National Hospital of Kandy, Kandy, LKA; 3 University Surgical Unit, National Hospital of Sri Lanka, Colombo, LKA

**Keywords:** anterolateral thigh free flap, facial reconstruction surgery, maxillary sinus tumor, squamous cell carcinoma (scc), total maxillectomy

## Abstract

Squamous cell carcinoma (SCC) of the maxillary sinus often necessitates extensive surgical resection followed by complex reconstruction and adjuvant therapy. Reconstructive approaches in head and neck surgery have advanced significantly over recent decades, with microvascular free flaps offering superior functional and aesthetic outcomes. We report the case of a 54-year-old male with moderately differentiated SCC of the right maxillary sinus, initially treated with right-sided maxillectomy in 2021 and reconstructed using a forehead local flap. Following adjuvant radiotherapy, the local flap failed within two months, resulting in a large maxillary defect and extensive post-radiation scarring. In January 2025, definitive reconstruction was performed using an anterolateral thigh (ALT) free flap in this fibrotic, irradiated field. Despite the technically challenging dissection, the reconstruction was successful, achieving satisfactory facial contour and proper wound healing. The case highlights the challenges of managing late-stage complications and the importance of free flap repair in facial reconstruction.

## Introduction

Malignant tumors of the nasal cavity and paranasal sinuses are quite uncommon, accounting for 0.2-0.8% of all human cancers. Squamous cell carcinoma (SCC) is the predominant histologic subtype and most frequently originates in the maxillary sinus (60-70%), followed by the nasal cavity (12-25%), ethmoid (10-15%), and other paranasal sites (1%) [1]. Incidence is higher in the Southeast parts of Asia, where long-term habits such as tobacco smoking and betel-quid chewing are prevalent risk factors [2]. SCC of the maxillary sinus typically occurs in middle-aged men who have been smoking or chewing betel for at least a decade [3]. Because early symptoms are nonspecific, many patients are diagnosed at an advanced stage, requiring extensive resection as well as adjuvant chemoradiotherapy [4]. Reconstruction after maxillectomy with cervical block dissection remains challenging, particularly in patients who have received postoperative radiation, as tissue fibrosis and poor vascularity increase the risk of flap failure. Microvascular free-tissue transfer has become the current standard for treating large, complex mid-face defects, with improved functional and aesthetic outcomes compared to regional flaps [5]. We report a case of moderately differentiated SCC of the maxillary sinus in a middle-aged man who initially underwent local flap reconstruction, which failed shortly after postoperative radiotherapy, resulting in a large maxillary defect. Later, the defect was successfully repaired using an anterolateral thigh (ALT) free flap. Despite the technically difficult dissection in the irradiated, scarred tissue, the reconstruction restored facial contour and achieved durable healing. This case illustrates the multidisciplinary approach and meticulous microsurgical techniques required for secondary facial reconstruction in a previously irradiated field.

## Case presentation

A 54-year-old male, an ex-smoker with a 10-pack-year history and a history of betel quid chewing, presented with a two-month history of right-sided facial swelling and pain, which had gradually worsened over the said period. Although the initial ultrasound scan (USS) of the infraorbital and buccal region suggested a hematoma, the oral and maxillofacial (OMF) team performed an incisional biopsy of the lesion through the oral cavity. Histology revealed moderately differentiated SCC of the right maxillary sinus. Then, contrast-enhanced computed tomography (CECT) of the head and neck was arranged, and it revealed histologically proven right-side maxillary sinus SCC with bony destruction and right-side cervical level I lymph node enlargement without brain metastases. After revealing the cancer of the maxillary sinus, the OMF team arranged the initial surgery to excise the tumor. Thereafter, right-sided maxillectomy combined with modified cervical block dissection, followed by forehead local flap reconstruction, was performed under general anesthesia in 2021. Histopathology confirmed moderately differentiated SCC infiltrating the deep and lateral margins, without lymphovascular invasion. Following the initial surgery, the patient underwent an adjuvant chemoradiotherapy regimen under the care of the oncology team at National Hospital Kandy. Although it was completed successfully, the patient developed radiotherapy-associated keratitis in the right eye and wound dehiscence of the flap reconstruction over the right infraorbital and buccal regions two months after the initial surgery. An attempt to revise the forehead flap was unsuccessful due to the severity of complications. Ophthalmology counselling was undertaken for right-sided eye keratitis, and a conjunctival patch graft and tarsorrhaphy were attempted but were unsuccessful, resulting in complete blindness in the right eye.

**Figure 1:**
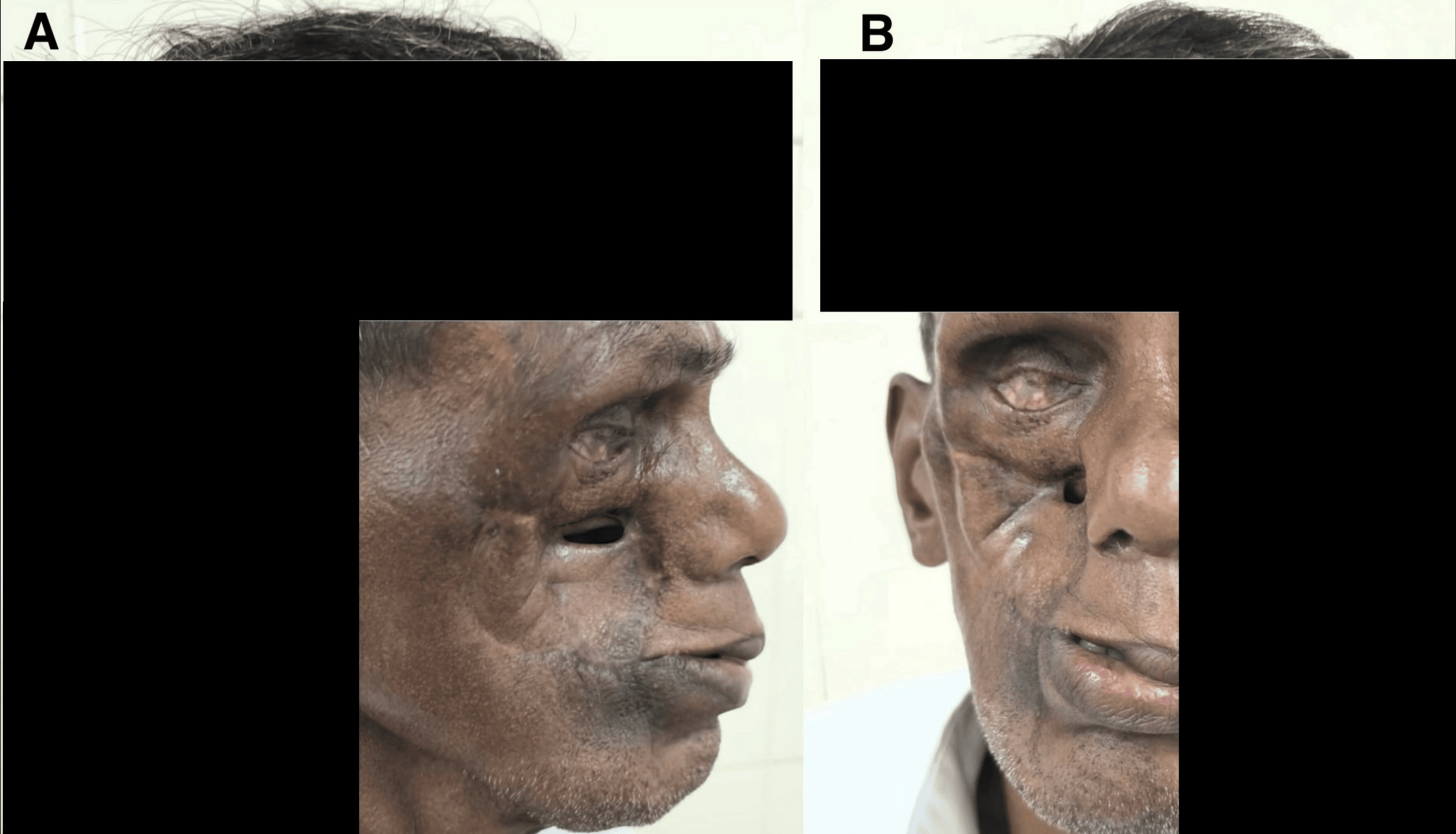
Preoperative images of the patient. A: lateral view; B: anterior view. Written informed consent to include this image in an open-access article was obtained from the patient.

Four years later, in 2025, the patient was referred for plastic reconstruction of the severely disfigured right side of the face. Before undergoing the procedure, it was confirmed that the patient was disease-free by a repeat CECT of the head, neck, and chest. On examination, there was right-sided cheek hypertrophic scarring with an infra-orbital opening that connected to both the middle meatus of the nasal cavity and the oral buccal mucosa (Figures 1A, 1B).

**Figure 2:**
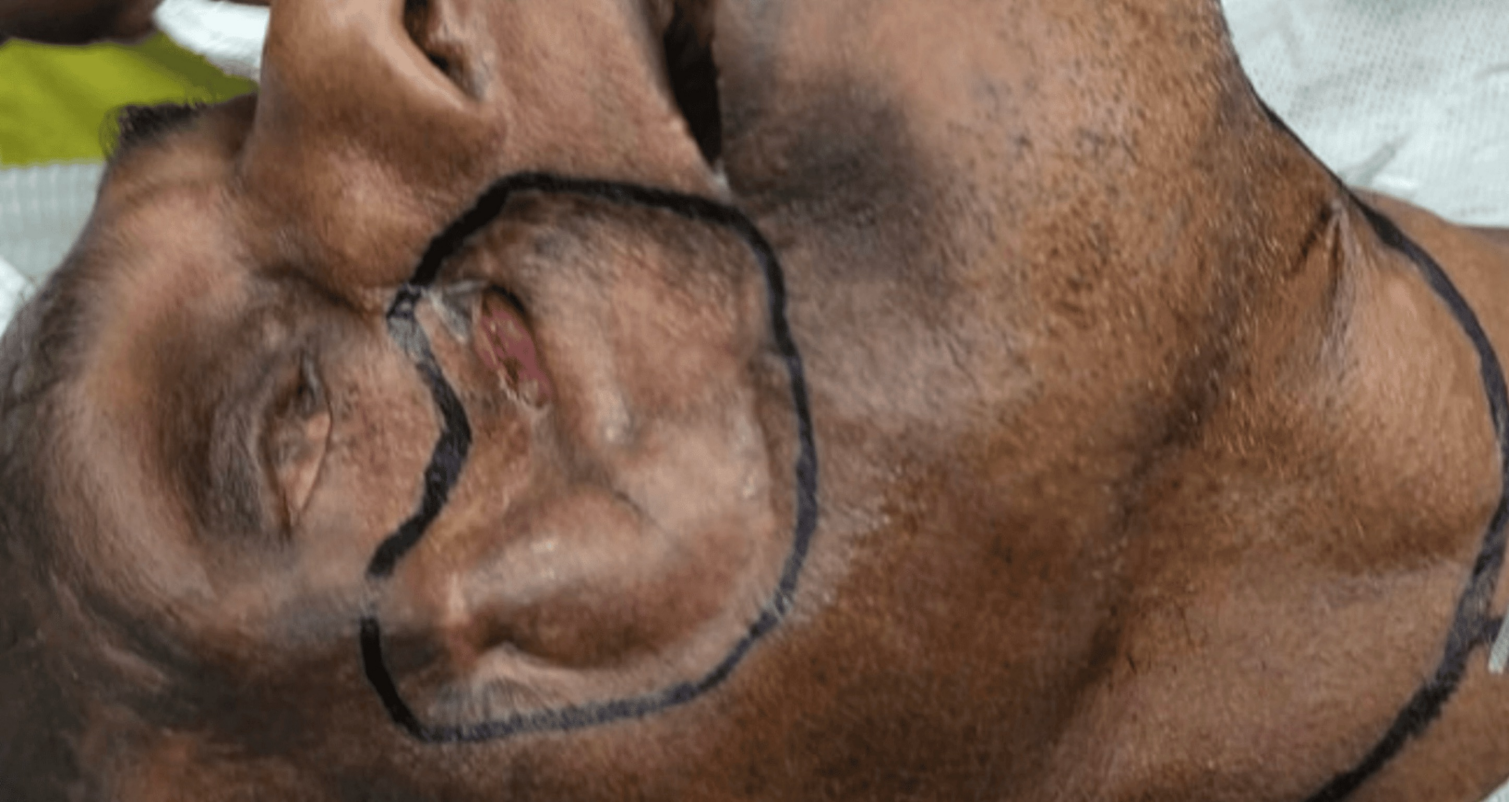
Perioperative marking of the incision sites. Written informed consent to include this image in an open-access article was obtained from the patient.

The fully consented patient underwent a free flap reconstruction to restore the right facial defect. The procedure involved the following steps. Incision sites were marked before commencing the procedure (Figure 2).

**Figure 3:**
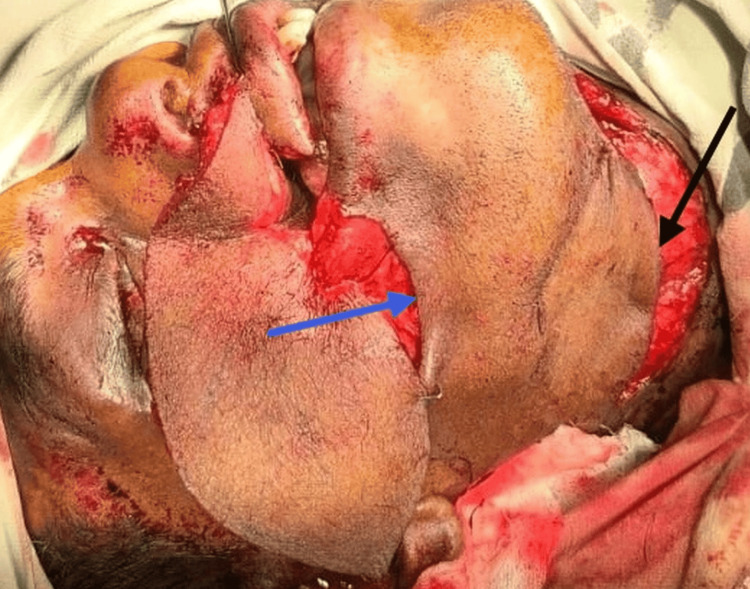
Perioperative image showing an anterolateral thigh flap over the defect site. The blue arrow shows the starting point of the subcutaneous tunnel, and the black arrow shows the endpoint of the tunnel. Written informed consent to include this image in an open-access article was obtained from the patient.

A tracheostomy was performed under local anesthesia before surgery for the management of the airway, as video-assisted laryngoscopy was unavailable and difficult intubation was anticipated. The airway was secured, and the patient was ventilated throughout the procedure. The initial incision was made over the infraorbital region as marked in the image, incorporating the previous scar tissues and the defect after injecting 1:200000 adrenaline for the reduction of bleeding. After excision of the infraorbital site, the neck incision was placed over the marked site, and the flap was raised. It was a difficult dissection because of the previous block dissection. After raising the neck flap, a subcutaneous tunnel was created over the mandible to the defect site. Thereafter, the ALT cutaneous free flap with the vascular pedicle was harvested from the left thigh. Though the initial plan was to anastomose the vascular pedicle to the facial artery, it was difficult to clear the facial artery of the adhesions. So, the artery pedicle was anastomosed to the right superior thyroid artery, and the vein was anastomosed to the right external jugular vein. Before starting the anastomosis, the ALT flap vascular pedicle was passed through the subcutaneous tunnel, which was created over the mandible (Figure 3).

**Figure 4:**
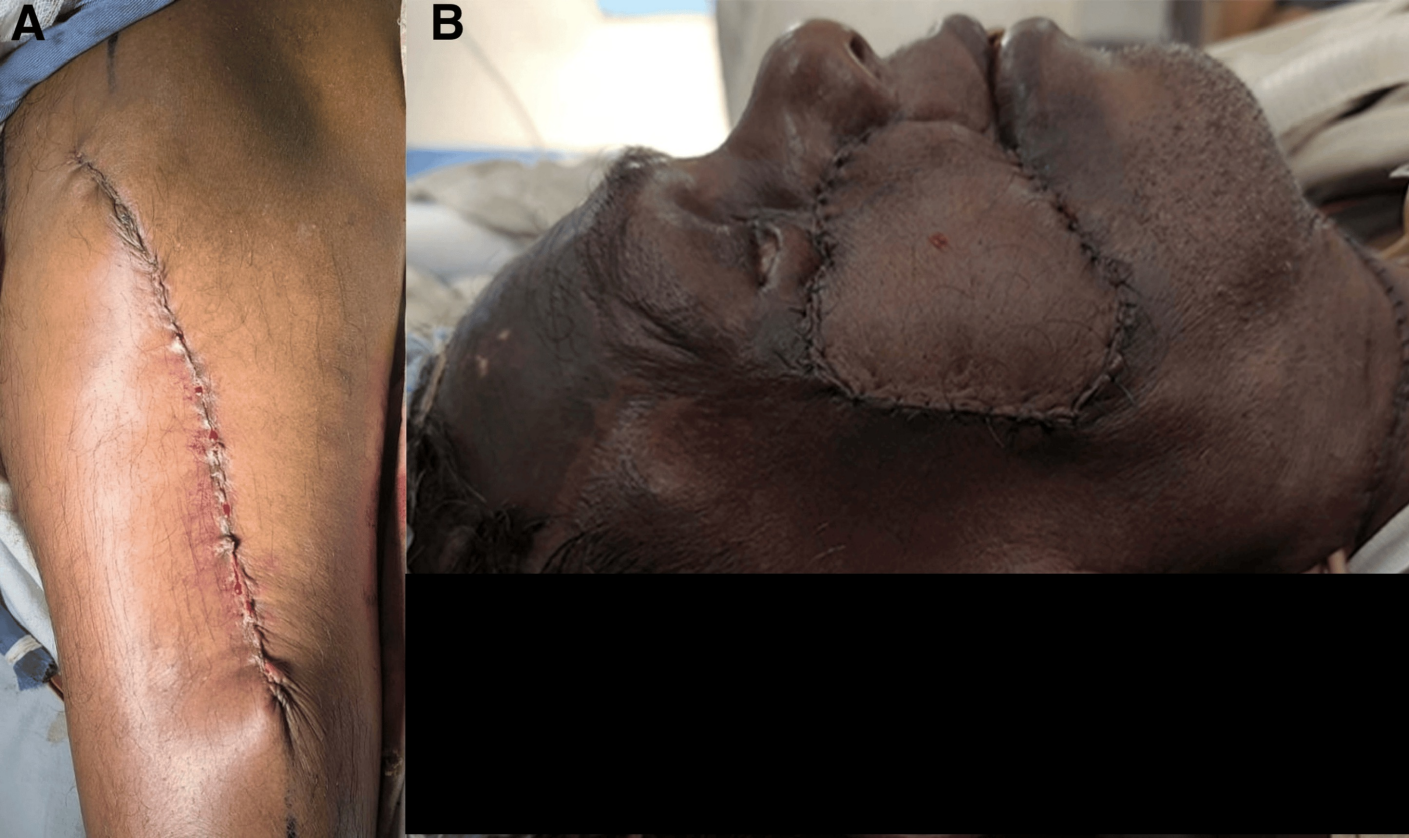
Images that were taken after completion of the surgery. A: donor site of the anterolateral thigh flap; B: lateral view of the reconstructed face. Written informed consent to include this image in an open-access article was obtained from the patient.

Then, anastomosis of the vascular pedicle was created as described above, with the assistance of a microscope and 10.0 polypropylene sutures. Adequate perfusion of the flap was visualized after the successful anastomosis. Then, the flap size was adjusted according to the defect size and sutured to the infraorbital defect site initially with subcuticular 5.0 poliglecaprone. Then, the skin was sutured carefully with 5.0 polypropylene while adjusting the size to match the defect size. The ALT donor site was primarily closed, and skin closure was achieved with 3.0 poliglecaprone. The images of the suture lines were taken at the end of the procedure (Figures 4A, 4B). A drain was inserted under the neck incision. Patient recovery was uneventful. A nasogastric tube was inserted intraoperatively for postoperative feeding purposes. In the postoperative period, flap viability was monitored for 48 hours using a Doppler machine. Aspirin (150 mg daily) was administered to maintain vascular patency. Tracheostomy care was given every two hours in the ward. The patient was mobilized on the following morning according to the enhanced recovery after surgery (ERAS) protocol. Early feeding was commenced. The drain tube was removed on postoperative day five. Intravenous antibiotics were administered perioperatively and continued till postoperative day five. Sutures were removed on postoperative day seven.

**Figure 5:**
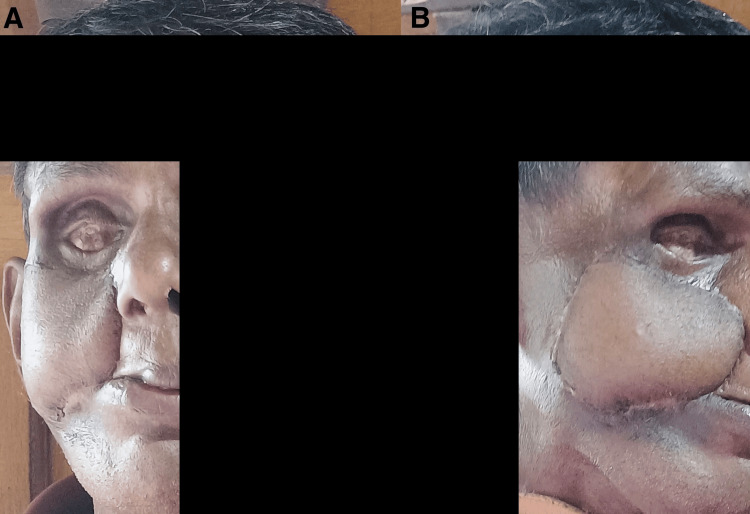
Images that were taken after removal of sutures and reversal of tracheostomy. A: anterior view; B: lateral view. Written informed consent to include this image in an open-access article was obtained from the patient.

The outcome of the facial reconstructive free flap repair was successful, and the tracheostomy reversal was done on postoperative day eight. The patient was discharged from the hospital on postoperative day eight. There were no major postoperative complications to mention. The patient returned to his daily routines from postoperative day 10 onwards (Figures 5A, 5B). The improvement in long-term quality of life has yet to be established after a 12-month follow-up with the aid of the Short Form-36 Health Survey [6]. 

## Discussion

This case highlights the complexities of managing SCC of the maxillary sinus, particularly the sequelae of chemo-radiotherapy, including wound breakdown, local flap failure, and severe disfigurement. Though the complete excision of the tumor was achieved, the patient was left with severe disfiguration of the face, which caused a tremendous mental breakdown and low self-esteem. Since the first forehead local flap reconstruction failed, and the operative field had already undergone extensive dissection, options for secondary reconstructive repair were limited. Therefore, microvascular free-tissue transfer is considered the gold standard for treating head and neck defects [5]. According to the currently available data, the first successful free flap reconstruction was reported in 1971 by Daniel & Taylor [7]. With fine refinements in microsurgical techniques, increased surgical expertise in the field, and improved instrumentation in the present day, the success rates of free flap reconstruction have pushed beyond 95% [8]. There are multiple donor sites available for extremity free flaps, including the ALT, fibula osteocutaneous, and superficial radial forearm fasciocutaneous free flaps [9]. The most common complications related to microvascular free flaps are surgical site infections, hematoma formation, seroma formation, and fistula formation [10]. Although the free flaps carry a considerable rate of common complications, which are around 47%, the major complications, such as flap necrosis, are reported in approximately 4% of cases [11]. In this case study, the patient was given only one antiplatelet agent (aspirin 150 mg daily) for five days without receiving any anticoagulation medication. However, the perioperative use of anticoagulation is much debated in free flap repair. Some studies have shown that there is no benefit from anticoagulation after microvascular surgery [12], whereas other studies have been neutral about the use of anticoagulants and antiplatelets [13,14]. High-quality randomized controlled trials are yet to be published to provide a proper conclusion to the above dilemma. The use of ALT free flap reconstruction demonstrates the importance of advanced surgical techniques in addressing complex defects. The challenges posed by prior radiation therapy, such as difficult vascular dissection, underscore the need for meticulous surgical planning and multidisciplinary coordination.

## Conclusions

Plastic and reconstructive surgery plays a vital role in restoring both function and appearance, not only in the region of head and neck oncologic resections but also throughout the body. It can yield excellent results when performed with careful planning and meticulous technique. This case demonstrates that ALT free flap reconstruction is an excellent and reliable option for complex maxillary defects, even after the patient had failed local flaps and adjuvant radiotherapy. Multidisciplinary planning and meticulous microvascular techniques are critical for successful surgery. The final verdict would be that the ALT flaps represent a versatile and dependable option for complex maxillary defects, even in patients with failed local flaps and prior adjuvant radiotherapy.
